# Remediation Strategies to Control Toxic Cyanobacterial Blooms: Effects of Macrophyte Aqueous Extracts on *Microcystis aeruginosa* (Growth, Toxin Production and Oxidative Stress Response) and on Bacterial Ectoenzymatic Activities

**DOI:** 10.3390/microorganisms9081782

**Published:** 2021-08-23

**Authors:** Zakaria Tazart, Maura Manganelli, Simona Scardala, Franca Maria Buratti, Federica Nigro Di Gregorio, Mountasser Douma, Khadija Mouhri, Emanuela Testai, Mohammed Loudiki

**Affiliations:** 1 Istituto Superiore di Sanità, Environment & Health Department, Viale Regina Elena, 299, 00161 Rome, Italy; zakaria.tazart@gmail.com (Z.T.); simona.scardala@iss.it (S.S.); franca.buratti@iss.it (F.M.B.); federica.nigrodigregorio@iss.it (F.N.D.G.); emanuela.testai@iss.it (E.T.); 2 Water, Biodiversity and Climate Change Laboratory, Phycology, Biotechnology and Environmental Toxicology Research Unit, Faculty of Sciences Semlalia, Cadi Ayyad University, Av. Prince My Abdellah P.O. Box 2390, Marrakech 40000, Morocco; mouhri@uca.ma (K.M.); loudiki@uca.ac.ma (M.L.); 3 Environmental Microbiology and Toxicology Research Unit, Polydisciplinary Faculty of Khouribga (FPK), Sultan Moulay Slimane University, Beni Mellal 23000, Morocco; douma_mountasser@yahoo.fr

**Keywords:** toxic cyanobacteria, anticyanobacterial activity, microcystins, cylindrospermopsin, Moroccan macrophytes, allelochemicals, bloom remediation, bacterial degradation activity

## Abstract

Increasing toxic cyanobacterial blooms in freshwater demand environmentally friendly solutions to control their growth and toxicity, especially in arid countries, where most drinking water is produced from surface reservoirs. We tested the effects of macrophyte allelochemicals on *Microcystis aeruginosa* and on the fundamental role of bacteria in nutrient recycling. The effects of *Ranunculus aquatilis* aqueous extract, the most bioactive of four Moroccan macrophyte extracts, were tested in batch systems on *M. aeruginosa* growth, toxin production and oxidative stress response and on the ectoenzymatic activity associated with the bacterial community. *M. aeruginosa* density was reduced by 82.18%, and a significant increase in oxidative stress markers was evidenced in cyanobacterial cells. Microcystin concentration significantly decreased, and they were detected only intracellularly, an important aspect in managing toxic blooms. *R. aquatilis* extract had no negative effects on associated bacteria. These results confirm a promising use of macrophyte extracts, but they cannot be generalized. The use of the extract on other toxic strains, such as *Planktothrix rubescens*, *Raphidiopsis raciborskii* and *Chrysosporum ovalisporum*, caused a reduction in growth rate but not in cyanotoxin content, increasing toxicity. The need to assess species-specific cyanobacteria responses to verify the efficacy and safety of the extracts for human health and the environment is highlighted.

## 1. Introduction

Several studies have shown increased frequency, intensity and duration of cyanobacterial blooms in aquatic ecosystems worldwide, due mainly to eutrophication and global warming [1]. Cyanobacterial blooms can produce a variety of compounds (e.g., cyanotoxins and taste and odor chemicals) that may have a negative impact on water quality and can represent a risk for human and animal health through drinking, recreational activities, consumption of contaminated food and feed items and irrigation [2,3,4]. Therefore, the control and prevention of cyanobacterial blooms and their toxicity, in terms of toxins production, is an important goal for monitoring water quality, drinking water supplies and prevention of adverse environmental and health impacts.

Many approaches to control harmful algae and cyanobacterial harmful algal blooms (CyanoHABs) have been proposed. These include filtration, UV and ultrasound in water treatment plants [5,6], artificial mixing and thermal destratification [7,8] and biological manipulations introducing selected fish and large zooplankton species in lake and/or reservoirs [1,9]. Most of these approaches, however, were primarily used as emergency solutions and have not always been successful while at the same time presenting drawbacks for ecosystem health. At present, H_2_O_2_ seems to be the most successful, even if it is advised to monitor the impact on nontarget species on a case-by-case basis [10].

Recently, the use of macrophyte allelochemicals has received increasing attention as an alternative to synthetic chemicals in CyanoHABs control, due to their assumed environmental safety and higher biodegradability [11]. Indeed, macrophytes are known to release allelochemicals by means of root exudation, leaching, volatilization and decomposition of plant residues in the water. Allelochemicals have been shown to efficiently reduce phytoplankton growth and algal biomass in eutrophic lakes, maintaining clear water [12,13].

Laboratory bioassay experiments using macrophyte extracts [14,15] or purified compounds to inhibit cyanobacterial growth [16,17,18,19] showed the effectiveness of macrophytes allelopathy in controlling cyanobacterial growth. In mesocosm and field experiments, treatment with different macrophytes gave rise to various degrees of inhibition of cyanobacterial blooms [20,21]. In one field experiment in a Chinese lake, Liu et al. [22] also showed that cocultivation of *Myriophyllum spicatum* not only dramatically reduced the density of *Microcystis* sp. but also led to an overall increase in eukaryotic phytoplankton diversity.

Even if phytoremediation seems unharmful for other phytoplankton species, studies on the effects on one of the main roles of the bacterial community in lakes, that is nutrient recycling through their ectoenzymatic activities [23], have never been carried out. Furthermore, most studies addressed only the effects on cyanobacterial growth, and scant and contrasting information is available on the effects of allelochemicals on cyanotoxin production, which is actually the main health hazard related to CyanoHABs [19,24,25,26]. Being limited and inconclusive, these results warrant further investigation.

As in many arid African countries where freshwater is scarce, the occurrence of CyanoHABs, mainly of *Microcystis aeruginosa,* has been reported in numerous drinking water reservoirs and natural inland waters in Morocco [27,28,29,30,31,32,33,34,35]. Owing to its geographical position in the Mediterranean hot spot area, Morocco has more than 600 species of macrophytes [36]. Previous studies [14,37] demonstrated that *M. aeruginosa* growth, Chlorophyll-a and carotenoid content are inhibited by aqueous extracts of *Ranunculus aquatilis*, a very common plant in Morocco, not previously studied for the purpose of remediation.

This study aims to (1) compare the potential algicidal activities of three additional Moroccan macrophytes (*Scirpus lacustris*, *Potamogeton natans* and *Cymodocea nodosa*) to that of *R. aquatilis,* (2) investigate the allelopathic effects of the most active one on growth and toxicity of *M. aeruginosa* by determining intra- and extracellular microcystins, (3) examine oxidative stress markers to better understand the allelopathic mechanisms, (4) study some ectoenzymatic activities of the *Microcystis* phycosphere (*Microcystis* and associated bacteria) as markers of important ecological functions and (5) preliminarily assess the response in growth and toxicity of other toxic species (microcystin and cylindrospermopsin producers) from different habitats and geographical areas. This paper considers for the first time the harmful cyanobacterial bloom control by allelochemicals from several points of view, including toxin production and associated bacterial communities, providing a more complete picture of the possible remediation activities, not limited to growth inhibition.

Our main results show the efficacy of aqueous extracts in controlling the density and toxicity of *M. aeruginosa*, without affecting the degradation activities of the bacterial community. However, they also show different responses of various cyanobacterial species, with an increase in toxin formation, stressing the need for species-specific studies. Further studies are also needed to identify the chemical compounds responsible for the allelopathic effects and their safeness for human and nontarget species health.

## 2. Materials and Methods

### 2.1. Cyanobacterial Strains

The non-axenic, colonial and monoclonal *M. aeruginosa* MCACt strain isolated from the Lalla Takerkoust eutrophic reservoir [14], a microcystin (MC) producer, was used to investigate the allelopathic efficacy of four macrophyte aqueous extracts. Subsequently, the most efficient extract was used to investigate the effects on *Microcystis* growth, physiology and toxin production.

The most efficient aqueous extract was also tested very preliminarily on the growth and toxicity of three filamentous cyanobacterial monoclonal non axenic strains: *Planktothrix rubescens* isolated from Flumendosa Lake, strain F10 (Italy [38]), MC producing, and two ANACC (Australian National Algae Culture Collection; CSIRO Tasmania) reference strains, CS1101 *Raphidiopsis raciborskii* and CS1034 *Chrysosporum ovalisporum*, both cylindrospermopsin producers [39].

### 2.2. Macrophyte Sampling and Extraction

The common water crowfoot (*Ranunculus aquatilis* L.) and the common club rush (*Scirpus lacustris* L.) were collected during their flowering period from Oukaimeden River (31°12′19″ N, 7°51′44″ W; Marrakech area) and Irriri river (30°93′75.7” N; 7°21′06.3” W; Ouarzazate area), respectively. The floating pondweed (*Potamogeton natans* L.) was collected from a small reservoir in Oukaimeden (31°12′31″ N, 7°51′06″ W; Marrakech area), while the seagrass (*Cymodocea nodosa* (Ucria) Asch.) was collected from a shallow part of the El Jadida Atlantic coast (33°15′38” N; 8°31′16” W).

The plant material was rinsed with distilled water to remove all debris and impurities then dried in the shade at room temperature (~25 °C). After that, the dried material was divided into small pieces and then powdered well with a grinder. Aqueous extracts of macrophytes were obtained from 20 g powder of macrophytes in 300 mL of sterile MilliQ (Merck KGaA, Darmstadt, Germany) for 24 H at 25 °C. After filtration with a GF/F (Whatman, Maidstone, England) filter to remove debris and fine particles, the obtained extracts were lyophilized and stored at −20 °C.

### 2.3. Comparison of Anticyanobacterial Activity of Different Macrophytes Extracts

The most efficient macrophyte against cyanobacterial growth was determined by an agar disc diffusion anticyanobacterial assay, with respect to the positive control of copper sulfate [40], and by assessing the minimum inhibitory concentration (MIC) and the minimum algicidal concentration (MAC) in microwell plates [37] (for details, see Supplementary Materials S1.1). The MIC represents the lowest extract concentration preventing cyanobacterial growth (bacteriostatic effect), while MAC represents the lowest extract concentration that induces 100% cell death of incubated cyanobacteria (bactericidal effect). The extract with the largest inhibitory area and the lowest MIC was the most bioactive one, used in the subsequent experiments.

### 2.4. Allelopathic Effect of the Most Active Aqueous Extract on M. aeruginosa Growth, Physiology and Toxicity

#### 2.4.1. Experimental Design

Three replicates of 200 mL of *M. aeruginosa* cultures in the exponential growth phase (2 × 10^9^ cells/L), cultured in BG11 medium (Fluka, Sigma-Aldrich, Buchs, Switzerland), were incubated with the MIC and half of the MIC of the most bioactive extract (hereafter MIC and MIC/2 conditions). We tested MIC/2 to address one of the problems in exporting microcosm experiment results to the field scale by reducing the amount of chemical compound used. The cultures inoculated with copper sulfate (at minimum inhibitory concentration) and untreated cultures were used as positive and negative controls (PC and NC), respectively. All cultures were maintained for 8 days in an incubator (25 °C, 2500 lux and 15:9 = light/dark), in the presence of oxygen. Samples were taken before the start of the experiment and every day (9.00 a.m.) to measure cell density, pigments (Chlorophyll-a, carotenoids) and pheophytin concentrations and every other day for the other parameters (bacterial abundance, microcystin concentration measured by immunoassay and oxidative stress markers). Samples to check for bacterial ectoenzymatic activities and for microcystin content by HPLC-DAD and LC-MS/MS analysis were taken at T0 after the addition of the different compounds (macrophyte extracts and copper sulfate) and T final.

#### 2.4.2. Cyanobacterial Density and Growth Rate Determination

*M. aeruginosa* cells were counted using a Malassez counting cell, and the growth parameters of each condition were calculated according to the equations of inhibition rate for each day (IR; Equation (1)) and mean growth rate (µ; Equation (2)):

(1) $$(\mathrm{IR}\;\left(\%\right)=\frac{\left(N_{c}-N_{S}\right)}{N_{c}}\times 100\;$$

(2) $$µ=\frac{\ln(N_{t}/N_{0})}{t}$$

where N_c_ and N_s_ are the cell densities (cells/mL) in the control and treatment samples, respectively.

N_0_ and N_t_ are the cell densities (cells/mL) at time 0 and after time t.

#### 2.4.3. Pigment Contents and Pheophytin Concentrations

Chlorophyll-a (Chl-a), carotenoid and pheophytin concentrations were determined using spectrophotometric methods according to Strickland and Parsons [41] and Siedlewicz et al. [42]. The absorbance measurements for Chl-a, carotenoids and pheophytin were recorded by a UV-vis spectrophotometer (HACH LANGE, DR 2800, Loveland, CO, USA) at wavelengths: 750, 665, 662, 645, 630 and 470 nm. The concentrations of the Chl-a and carotenoids were calculated according to Lichtenthaler and Wellburn [43] and pheophytin according to Lorenzen [44].

#### 2.4.4. Antioxidant Response of *M. aeruginosa*

The total protein content [45], malondialdehyde (MDA) concentration and the activities of the antioxidant enzymes superoxide dismutase (SOD) and catalase (CAT) were measured to investigate the antioxidant response of *M. aeruginosa* cells and to have an insight into the possible mechanism of the anticyanobacterial effect. SOD activity was assayed according to Beauchamp and Fridovich [46]; CAT activity was assayed according to the method of Rao et al. [47], and MDA content was determined colorimetrically according to Esterbauer and Cheeseman [48]. Methods were slightly modified by the original protocols [49] (Supplementary Materials S1.2.1).

#### 2.4.5. Cyanobacteria Morphology and Bacterial Abundance

One hundred microliters of formaldehyde-fixed samples (final concentration of 4%) were stained with SyberGreen (Invitrogen, Waltham, MA, USA), a nucleic acid stain, at 1× concentration and then filtered onto 0.2 µm black membrane filters (25 mm diameter, GTBP, Millipore, Merck KGaA, Darmstadt, Germany). The dried filters (room temperature, in the dark) were mounted on the slides with the antifade solution according to Patel et al. [50].

The cyanobacteria morphology was observed by confocal scanning microscopy (Leica TCS SP2, Leica Microsystems GmbH, Wetzlar, Germany), under 633 ex/650–750 em for Chl-a autofluorescence and 488 ex/500–535 em for SyberGreen-stained DNA. The bacteria were counted using at least 20 frames or 200 cells per sample, from 2 replicate slides, at 1000× magnification under a right fluorescence microscope (Olympus, BX51, Olympus corporation, Tokyo, Japan), with an FITC filter.

#### 2.4.6. Cyanotoxins Analysis

*Microcystis aeruginosa* produces mainly MCs, a class of hepatotoxins with more than 250 variants [51] (Figure S1, Table S1, Supplementary Materials S1.2.2). Three methods were used for comparison and efficiency. The immunological semiquantitative ELISA test was used to see the temporal trend in the production of toxins (as a sum of the different variants) during the experiment, as it is sensitive and requires a very small volume. At T0 and T final, when larger volumes were available, HPLC-DAD and LC-MS/MS were also used to determine and quantify the various MC congeners produced in the different experimental conditions.

Each sample was divided into two aliquots, one to determine extracellular dissolved MCs (GF/C discs filtrate) and the other to determine the total toxin concentration, after three freeze–thawing cycles. Intracellular toxin was obtained by subtracting extracellular content from total MC concentration.

For ELISA, 2 mL water samples were filtered by membrane filters (0.22 mm, Millex-GV, Millipore, Merck KGaA, Darmstadt, Germany) just before the analysis and then analyzed with a commercially available Envirologix QuantiPlate^TM^ Kit for Microcystins High Sensitivity (Envirologix, Portland, ME, USA), following the instructions of the producer, by measuring the absorbance at 450 nm (Wallac Victor2 spectrofluorometer, Perkin Elmer Inc., Waltham, MA, USA). The limit of quantification (LOQ) was 0.15 µg/L. Data are expressed as MC-LR equivalent.

For HPLC-DAD and LC-MS/MS, all samples (total and filtrate) (150 mL at T0 and 60 mL at T final) were filtered through GF/C filters to get rid of cell debris, acidified with 0.1% formic acid, purified and concentrated through ODS SPE cartridges. Briefly, the cartridges were preconditioned with 4 mL of MeOH followed by 4 mL of ultra-pure-grade acidified water. Samples were passed through the cartridge and washed with 20% MeOH (4 mL), then dried under vacuum before being eluted by 6 mL of MeOH (recovery percentages ≥85%).

HPLC-DAD was used only for total (intracellular plus extracellular) samples, further concentrated to 0.5 mL. Identification and quantification of the variants MC-LR, MC-LW, MC-RR, MC-YR, MC-LF were obtained using the method described by Buratti et al. [52] (for details, see Supplementary Materials S1.2.2). A calibration curve for each variant was prepared with at least 5 or 7 concentrations within the range 0.5–100 μM (R^2^ > 0.99 for all tested congeners).

For LC-MS/MS, separation and identification of the MC variants (MC-RR, MC-YR, MC-LR, MC-LA, MC-LW, MC-LF, MC-LY, [d-Asp3]-MC-RR, [d-Asp3]-MC-LR, MC-HtyR, MC-HilR, MC-WR) were performed according to Nigro Di Gregorio et al. [53] (for details, see Supplementary Materials S1.2.2). Quantification of the detected MCs was performed by the external standard procedure, referring to calibration straight lines (correlation coefficient R^2^ = 0.97–0.99). The limit of quantification was 5–25 pg injected on the column.

Analytical standards of MC congeners tested, MC-RR, MC-YR, MC-LR, MC-LA, MC-LW, MC-LF, MC-LY, [d-Asp3]-MC-RR, [d-Asp3]-MC-LR, MC-HtyR, MC-HilR, MC-WR, with purity ≥95%, were obtained from Enzo Life Sciences (Farmingdale, NY, USA).

#### 2.4.7. Ectoenzymatic Activities

Ectoenzymatic activities (cell-surface-bound and periplasmic) were determined to check on the degradation activity of the bacterial/cyanobacterial community according to the method described by Manganelli et al. [54]. Briefly, leucine aminopeptidase, alkaline phosphatase, α-glucosidase, β-glucosidase and *N*-acetyl-β-d-glucosidase were measured at saturating substrate concentrations using analog substrates covalently linked to a fluorophore (4-Methylumbelliferyl (4-MUF) or 7-amido-4-methylcoumarin hydrochloride (7-AMC), Sigma-Aldrich, Merck KGaA, Darmstadt, Germany) [55]. The final concentrations used were 250 µM for all the substrates used (L-leucine-7-AMC; 4-MUF-phosphate; 4-MUF-α-galactoside; 4-MUF-β-galactoside and 4-MUF-*N*-acetyl-β-d-glucosaminide, Sigma-Aldrich). Fluorescence was measured at 355/460 nm excitation/emission (Wallac Victor2 spectrofluorometer, Perkin Elmer Inc., Waltham, MA, USA). Quantification of 4-MUF and 7-AMC was achieved by calibration with standard solutions in each of the same media of the experiments (BG11 and BG11 plus CuSO4 and plant extracts).

### 2.5. Effects of the Most Active Aqueous Extract on Other Cyanobacterial Species

Triplicate 4 mL cultures at exponential phase for each of the filamentous species, with and without macrophyte aqueous extract (control), were set up to look only at growth inhibition and toxin production. Cyanobacterial density and total toxin concentration were measured at T0 and T final, after 10 or 8 experimental days. To measure cell density, 50 µL of fixed cultures were diluted to 2 mL with sterile MilliQ and filtered onto 0.2 µm membrane filters (see Section 2.4.5). Because filamentous cyanobacteria in lab cultures produce very long filaments, 30–40 pictures were taken in autofluorescence at 10× magnification, and then the total length of filaments per field from the binarized images were measured with the open-source software ImageJ 1.53 g (https://imagej.nih.gov/ij/, accessed on 17 August 2021). Total toxin concentrations were measured by ELISA with the Envirologix QuantiPlate^TM^ Kit for Microcystins (see Section 2.4.6) and Eurofins Abraxis ELISA for cylindrospermopsin (Eurofins Abraxis, Warminster, PA, USA).

### 2.6. Data Analyses

All experiments were conducted in triplicate, and statistical analyses were performed using analysis of variance (two-factor ANOVA with Tukey’s test). The results are expressed as the mean of triplicate determinations ± standard deviation (SD). The confidence limits used in this study are based on *p* < 0.05, *p* < 0.01 and *p* < 0.001. The software SigmaPlot version 12.5 (Systat Software, Inc., Chicago, IL, USA) was used for statistical analysis.

## 3. Results

### 3.1. Comparison of Anticyanobacterial Activity Shown by Different Macrophyte Aqueous Extracts

The anticyanobacterial activity of the four macrophyte aqueous extracts was evaluated qualitatively using the disk diffusion method and quantitatively by the broth microdilution method in 96-well plates (Figure S2 and Table S2). The results show that all tested macrophytes inhibited *M. aeruginosa* growth. The most efficient was the extract of *R. aquatilis*, with zones of inhibition greater than 36 mm, followed by the marine macrophytes *C. nodosa* (32 mm) > *S. lacustris* (31 mm) > *P. natans* (30 mm).

The minimum inhibitory concentrations (MICs) and minimum algicidal concentrations (MACs) confirmed that *R. aquatilis* was the most active, with MIC and MAC values of 13 and 25 mg/mL (Table S2). The MICs of the other macrophytes ranged from 25 to 50 mg/mL (in the order *P. natans* = *C. nodosa* < *S. lacustris*), with MACs only slightly higher (50–100 mg/mL, in the same order).

The extract of *R. aquatilis* was used in the subsequent experiments.

### 3.2. Allelopathic Effect of R. aquatilis Aqueous Extract on M. aeruginosa and Their Associated Bacteria

#### 3.2.1. Growth Inhibition

**Figure 1:**
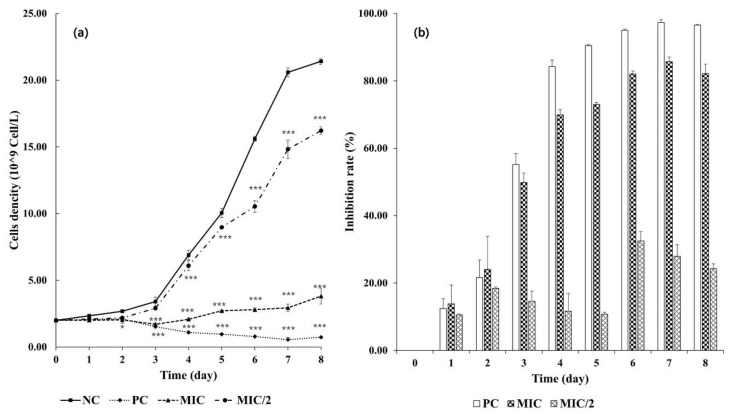
Effect of *R. aquatilis* aqueous extracts on the growth of *M. aeruginosa*. (**a**) cell density in 10^9^ Cell/L and (**b**) inhibition rate in percentage. Each value is the mean ± SD of three replicates. * *p* < 0.05 and *** *p* < 0.001 indicate significant differences between the treatments and the negative control (one-way ANOVA) in (**a**). NC: negative control; PC: positive control; MIC: minimal inhibitory concentration and MIC/2: half MIC concentration.

The cell density and inhibition rate of *M. aeruginosa* treated by different concentrations of *R. aquatilis* extract are presented in Figure 1.

In the control, *M. aeruginosa* grew rapidly, and cell density reached 21.41 × 10^9^ cells/L after 8 days of culture. In the MIC/2 treatment (6.5 mg/mL of *R. aquatilis* extract), *M. aeruginosa* growth was slightly but significantly inhibited from Day 4 (*p* < 0.001), with an inhibition rate (IR) of 11.61% that increased to 24.28% at the end of the experiment. In the MIC treatment (13 mg/mL of *R. aquatilis* extract), cell density was significantly lower than the control starting from Day 3 (IR = 49.85%). The highest inhibition rate (82.18%) was attained on Day 8 when the culture became transparent (with aggregation of the cells in the bottoms of the flasks), indicating a persistent inhibition effect. Copper sulfate (0.003 mg/mL) exerted the strongest effect (*p* < 0.001). No *M. aeruginosa* growth was observed from Day 1 when the cells actually started to decrease. Results on the *M. aeruginosa* parameter from the positive control will not be mentioned hereafter, as they were always consistent with no cyanobacterial growth.

#### 3.2.2. Effect on Pigments and Pheophytin Content of *M. aeruginosa*

**Figure 2:**
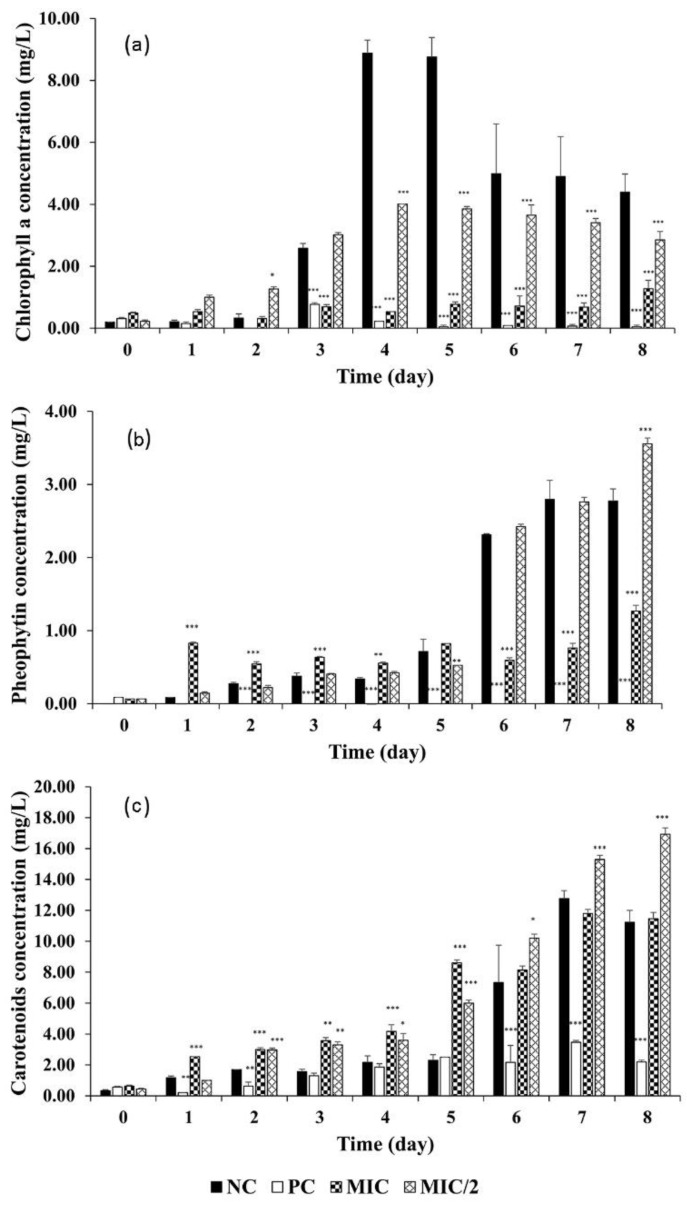
Effect of *R. aquatilis* aqueous extracts on the *M. aeruginosa* pigment contents. (**a**) Chl-a, (**b**) pheophytin, (**c**) carotenoids. Each value is the mean ± SD of three replicates. For the pigment concentrations, * *p* < 0.05, ** *p* < 0.01 and *** *p* < 0.001 indicate significant differences compared to the untreated culture (one-way ANOVA).

In all the treatments, Chl-a content was significantly affected with respect to the control, where it increased steadily to a maximum of 8.88 mg/L at Day 4 to reach 4.40 mg/L at the end of the experiment (Figure 2a). In the MIC/2 treatment, Chl-a concentration followed the same temporal pattern, but the maximum (4.05 mg/L, *p* < 0.001) and the level reached at the end of the experiment (2.85 mg/L, *p* < 0.001) were 1/2 the concentrations in the control group. In the MIC treatment, Chl-a reached a very low maximum concentration of 1.28 mg/L at the end of the experiments.

Pheophytin concentration in the control and the MIC/2 increased only from Day 4 until the end, reaching a significantly higher value in the MIC/2 (3.56 mg/L vs. 2.77 mg/L). In the MIC treatment, pheophytin was present from the very beginning of the experiment at a concentration higher than in the control (*p* < 0.001) until Day 4. It then continued to grow up to 1.27 mg/L at the end of the experiments, but due to a much lower cell density, it was significantly lower than the control (Figure 2b).

The carotenoid pigments continuously increased over the experimental time, with values significantly higher in the MIC and the MIC/2 than in the control (*p* < 0.05 and *p* < 0.001) until Day 5. In the following days, carotenoids reached a final concentration of 16.93 mg/L in the MIC/2, higher than the concentration of ~11 mg/L in the control and MIC (Figure 2c).

#### 3.2.3. Response of *M. aeruginosa* Cells to Oxidative Stress

**Figure 3:**
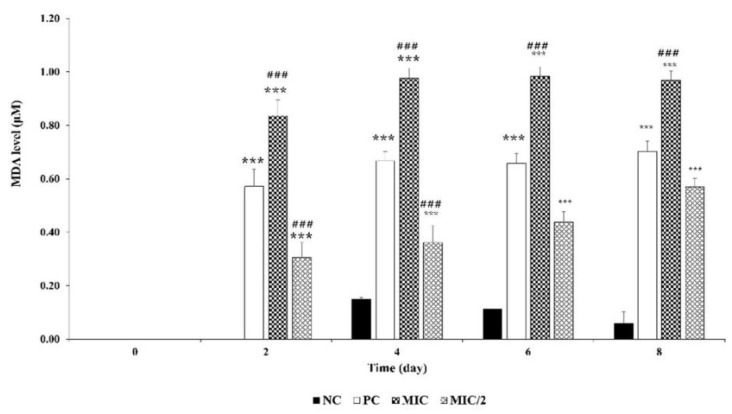
Effect of *R. aquatilis* aqueous extracts on MDA level of *M. aeruginosa* cells. Each value is the mean ± SD of three replicates. *** *p* < 0.001 indicate significant differences compared to the untreated culture and ### *p* < 0.001 indicate significant differences compared to the positive control. T0 < LOD.

*M. aeruginosa* exposed to the aqueous extract showed a significant dose-dependent response to oxidative stress from the beginning of the experiment. MDA was detected in the control only after 4 days, with consistently low concentrations (Figure 3). In the treated systems, MDA was detected from Day 2. In the MIC/2, it increased from 0.31 µM to 0.57 µM, and in the MIC, it was constant around 0.90 µM.

**Figure 4:**
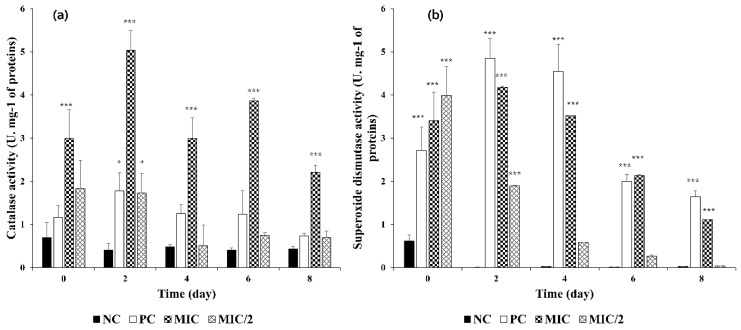
Effect of *R. aquatilis* aqueous extracts on the *M. aeruginosa* enzymatic activities. (**a**) catalase activity and (**b**) superoxide dismutase activity. Each value is the mean ± SD of three replicates. * *p* < 0.05 and *** *p* < 0.001 indicate significant differences compared to the untreated culture.

CAT activity in the control remained stable below 1 U∙mg^−1^ proteins, while SOD activity was detected only on the first day of the experiment (0.6 U∙mg^−^^1^ proteins) (Figure 4).

In the MIC/2, the highest values were reached from the very first day, with CAT at 1.8 and SOD at 4.01 U∙mg^−1^ proteins, then decreased constantly to very low levels. In the MIC, the activities were higher and without a clear trend. The highest CAT activity was detected after 2 days of exposure, at 5 U mg^−1^ proteins, then it fluctuated between 2 and 4 U mg^−1^ proteins. Analogously, SOD activity was high at Days 2 and 4 (around 4–5 U∙mg^−1^ proteins) and slightly decreased up to Day 8.

#### 3.2.4. Effect on Morphology of *M. aeruginosa* and Bacterial Community

**Figure 5:**
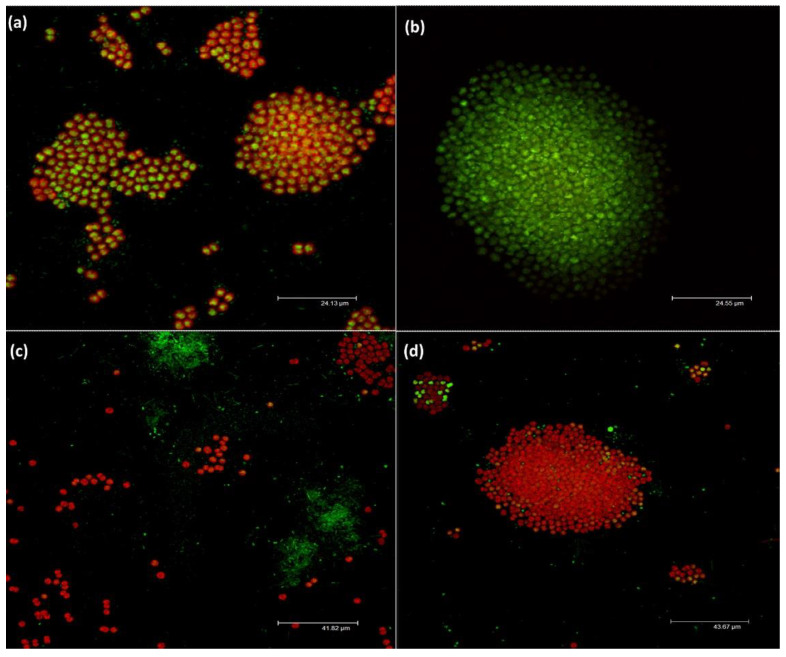
Confocal laser scanning micrographs of *M. aeruginosa* and bacterial community, labeled with SybrGreen at the end of the experiments in the different treatments. (**a**) NC, (**b**) PC, (**c**) MIC and (**d**) MIC/2. Red: pigment autofluorescence; SybrGreen stained DNA fluorescence.

At the end of the experiment, the morphology of *Microcystis* colonies and the distribution of bacteria around and inside the colonies were observed after SybrGreen staining by confocal scanning microscopy. The morphology of the colonies was clearly affected by the different treatments. In the control (Figure 5a) used to set up the conditions of image acquisition, the colonies looked healthy, with a strong fluorescence of the pigment and the DNA. Free bacteria were homogenously distributed in the field, and attached bacteria were visible between the cells on top of the colonies.

The shape of the colonies in the positive control (Figure 5b) was completely different, with cells no longer autofluorescent and aggregated in collapsed colonies; furthermore, copper sulfate had a toxic effect on bacteria too, keeping their density very low (see below). The *M. aeruginosa* cells in the MIC (Figure 5c) also appeared unhealthy, as shown by the very low signal in the green channel. No typical colonies were observed (Figure 5c), and only dispersed *Microcystis* cells appeared, surrounded by large aggregates of bacteria. In the MIC/2 (Figure 5d), few small healthy colonies were present, and there were less visible bacterial aggregates when compared to the MIC treatment conditions.

**Figure 6:**
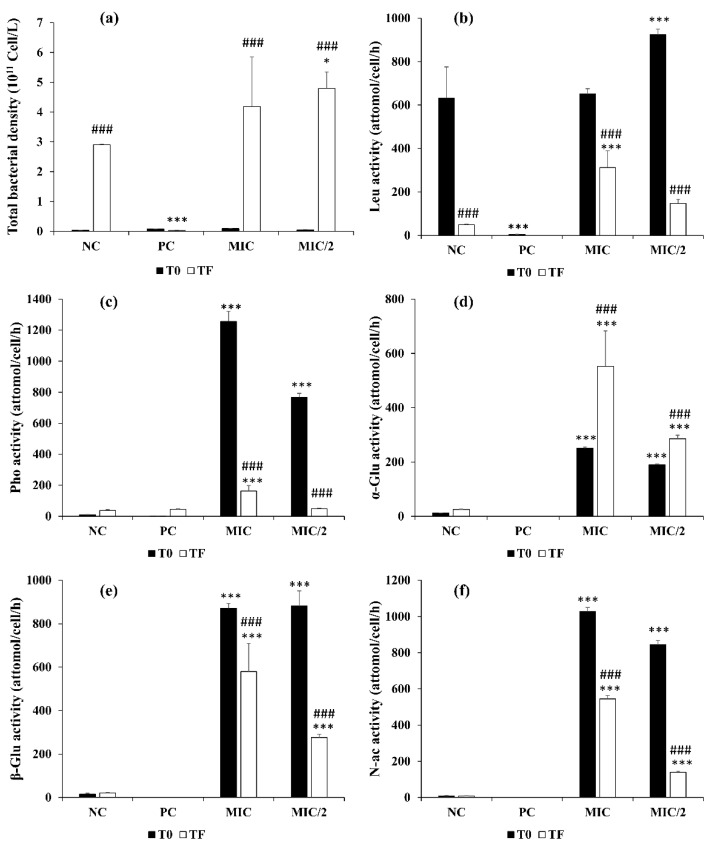
Effect of *R. aquatilis* aqueous extract on bacterial density and ectoenzymatic activities in the *M. aeruginosa* cultures in different treatments groups, at T0 (T initial) and TF (T final). (**a**) bacterial density, (**b**) leucine amino-peptidase, (**c**) alkaline phosphatase, (**d**) α-glucosidase, (**e**) β-glucosidase and (**f**) *N*-acetyl-β-d-glucosidase activities. Each value is the mean ± SD of three replicates. * *p* < 0.05 and *** *p* < 0.001 indicate significant differences compared to the untreated culture in the same time (initial or final); ### *p* < 0.001 indicate significant differences between T0 and TF in each treatment group.

Figure 6a shows the effect of the extract (MIC and MIC/2) and copper sulfate (PC) on the abundance of bacteria at T0 and T final. While bacterial density was strongly reduced by copper sulfate, it increased significantly in the control and in the cultures treated with *R. aquatilis* extracts (*p* < 0.001). At the end of the experiment, bacterial density was 4.19 × 10^11^ cell/L in MIC and 4.79∙10^11^ cell/L in the MIC/2, this latter value being significantly higher than 2.90∙× 10^11^ cell/L in the control (*p* < 0.05).

In the positive control, the ectoenzymatic activities were almost undetectable (Figure 6b–f). On the contrary, the presence of the aqueous extract immediately stimulated the activities in a significant way. All the enzymes except leucine-aminopeptidase at T0 were significantly more active than in the control by two to three orders of magnitude (*p* < 0.001). Leucine-aminopeptidase was about 600 attomole/cell/h in both the MIC and the control vs. a significantly higher value of 1000 attomole/cell/h in the MIC/2. However, the other activities were negligible in the control. α-, β- and *N*-acetyl-β-d-glucosidase were very similar in the MIC and the MIC/2, while alkaline phosphatase in the MIC was almost twice as much as in the MIC/2. At the end of the experiment, only α-glucosidase significantly increased in both the MIC and the MIC/2 (*p* < 0.001), while the other activities decreased, even if at a level even higher than in the control.

#### 3.2.5. Effects on Toxin Production

Intracellular MCs, as measured by the semiquantitative analysis of ELISA test, paralleled the temporal trend shown by cell density in the control, the MIC and the MIC/2. Indeed, MC-LReq correlated to cell density (n = 4, *p* < 0.01) in each of these three systems. Consistently, the highest MC-LReq was measured in the control (Table 1). In the positive control, the toxin concentration was the lowest. The extracellular values were always below the LOD (0.15 µg/L).

T0 and T final values measured by LC-MS/MS, as the sum of the different congeners, were in good agreement with the ELISA values. The analysis on the concentrated samples showed that the extracellular fraction was negligible in all samples, except in the positive control, where the majority of the very low toxin concentration was in that fraction, as expected, due to the disruptive action of copper sulfate on cells. The results expressed as the sum of different congeners in total samples at T final with HPLC-DAD were consistent only in the control (14.40 µg/L), whereas much higher values were obtained in the samples exposed to the macrophyte extract. The cell quota based on LC-MS/MS values was very similar between the control and the MIC (Table 1) but was significantly lower (*p* < 0.05) in the MIC/2 and in the PC.

Among the 12 MC congeners investigated, 5 MC variants were detected, namely: MC-LR as the most abundant, followed by MC-RR, -YR, and traces of -[d-Asp3]-MC-LR and -LW at T final (Figure S3). MC-LR was the most abundant in the control at T0 (55%) and increased its relative abundance up to 64%, while MC-RR decreased from 28% to 19%, and MC-YR remained constant at 16% (Figure S3a,b). The relative abundance of congeners in the MIC and the MIC/2 was quite similar (Figure S3c,d): MC-LR was ~50%, MC-YR was 15% and MC-RR was ~30%, with a very small percentage of -LW in the MIC and -[d-Asp3]-MC-RR in the MIC/2.

HPLC-DAD gave comparable results in the control (with an extra congener, MC-LW identified), but results obtained from the MIC and the MIC/2 were almost an order of magnitude higher.

### 3.3. Allelopathic Effect of R. aquatilis Aqueous Extract on Growth and Toxicity of CS1101 R. raciborsky, CS1034 C. ovalisporum and P. rubescens F10

The *R. aquatilis* aqueous extract also exerted allelopathic effects on the two cylindrospermopsin-producer cyanobacteria, causing a decrease in cell number, as shown by a final density 18 and 4.6 times lower in the treated *Raphidiopsis* and *Chrysosporum* samples, respectively, and their negative growth rates. However, the cylindrospermopsin concentration was only 2.3 times lower in *Raphidiopsis*, and no effect was observed in *Chrysosporum*, where the absolute toxin level was unaffected, meaning that in both cases, cell quota was increased (Table 2).

When *P. rubescens* was tested, the extract reduced growth rate in the MIC compared to the control (0.07 d^−1^ vs. 0.14 d^−1^), while the final cell quota in the exposed cells was not different from the control (Table 2).

## 4. Discussion

We investigated the inhibitory and algicidal activities of freshwater and marine macrophyte extracts from different Moroccan water bodies as a remediation strategy to control toxic cyanobacterial growth in water bodies. In order to verify the feasibility and the usefulness of this kind of remediation approach, we followed a series of parameters on cyanobacteria and on the associated bacterial community. Going beyond the study of cyanobacterial growth, as most cases found in the literature, is extremely important, as, whenever a remediation measure is considered, it should be effective against the noxious agent. At the same time, however, it must be “safe” for the ecosystem in general terms so that the possible “adverse effects” are also taken into account. In the specific case of CyanoHABs, no toxin production should be stimulated and, at the same time, no effects on nontarget organisms should be observed. We started to look at some of these aspects for the first time.

The aqueous extracts of all tested aquatic plants were potentially effective in suppressing the algal growth of *M. aeruginosa*. Among those tested, *R. aquatilis* extract was the most effective.

In the batch experiment, *R. aquatilis* aqueous extract was proven to control the density of *M. aeruginosa* in the same range of other macrophytes (10–25 mg/mL) with a comparable inhibition rate (75–98%) [14,56,57]. However, the exposure of *M. aeruginosa* to half of the minimum inhibitory concentration was not proportionally effective (82% vs. 24.3%), suggesting that there is no linear response between aqueous extract concentration and inhibition rate.

This feature is common to other parameters, namely pigments, MDA, CAT and SOD, revealing that oxidative stress was induced in *M. aeruginosa* exposed to *R. aquatilis* extract in a concentration-dependent manner, although not linearly.

The MIC treatment significantly lowered the concentration of Chl-a with respect to the control, consistent with density, as observed in other studies (e.g., [15,58]). The significantly higher pheophytin in the samples treated with the MIC from the first day and the positive correlation between Chl-a and pheophytin (r = 0.752, n = 9, *p* < 0.05) suggests that the *R. aquatilis* extract did not affect Chl-a synthesis but triggered its degradation. This seems confirmed by the per cell concentration of Chl-a and pheophytin, both significantly higher in the MIC than in the control (Figure S4a,b). In the treatment with the MIC/2, where Chl-a decreased as well, this is not that clear, as the effect is shifted in time, and a consistent increase in pheophytin (total and per cell) was observed only at T final.

Carotenoids, whose main function is to protect Chl-a against photo-oxidation [59], increased significantly with respect to the control, both as total content and as cell quota (Figure 2 and Figure S4c), in line with previous reports [60]. Because carotenoids act as antioxidants to scavenge free radicals [61], the synthesis of carotenoids induced by exposure to *R. aquatilis* extract could be one of the detoxification processes, activated by *M. aeruginosa*.

The induction of oxidative stress was also clearly demonstrated by the concentration and time-dependent increase of MDA, a biomarker of membrane lipid peroxidation in cyanobacteria and other photosynthetic organisms [62,63]. The increase in lipid peroxidation, causing cell membrane damage, is consistent with growth inhibition and demonstrates that allelochemicals are able to compromise membrane integrity, ion channel imbalance and alterations in membrane permeability [56,64,65]. Similar results were reported by Li et al. [56] and Yuan et al. [15] on *M. aeruginosa* exposed to extracts of *Sagittaria trifolia* and *Spartina alterniflora*.

Finally, the induction of CAT and SOD activities, antioxidant enzymes involved in the scavenging and conversion of excess H_2_O_2_ to water and oxygen, is further confirmation that *M. aeruginosa* experienced oxidative stress, but the results in the two treatments, MIC and MIC/2, are not comparable.

Cyanobacteria are equipped with an efficient antioxidant system to counteract the effects of increasing ROS concentration [15,56]. However, different strains can have different threshold values, and one factor could be related to the potential toxicity of the strain. A recent study [66] showed that when a strong oxidizing agent such as H_2_O_2_ is used for controlling CyanoHABs at lethal concentrations (i.e., 2–3 orders of magnitude higher than those naturally occurring in a lake surface), nontoxic strains are favored. The authors of the study showed that a *Microcystis* toxic strain did not express enzymes involved in the reduction of H_2_O_2_ genes (e.g., SOD and CAT) before being exposed to the oxidizing agent. On the contrary, in a nontoxic strain and in the nontoxic mutant of the same strain CAT and SOD genes were expressed also before exposure to H_2_O_2_, which accounted for the degradation of the high level of applied H_2_O_2_. This is an interesting finding for the purpose of controlling toxic cyanobacterial blooms. It suggests that by applying aqueous extract at minimum inhibitory concentration, a concentration purposely intended to inhibit the growth of *M. aeruginosa*, nontoxic strains could be favored vs. toxic ones.

Once the effectiveness of *R. aquatilis* extract was proven in inhibiting *M. aeruginosa* growth and the induction of oxidative stress in the cyanobacterial cells, we verified the effects of the extract on the potential toxicity associated with cyanotoxin production. Indeed, although the cell density was reduced, the amount of toxin per cell could have been increased as a defensive response to the treatment. Toxic cyanobacteria have shown more than one reaction under stress, and both intracellular and extracellular roles have been proposed for cyanotoxins. In the former case, they could be a further protective strategy for the cell during oxidative stress, and in the latter, they could act as an infochemical once released by lysed cells, but a univocal role/function of cyanotoxins has not yet been fully elucidated [67,68].

We measured the level of toxins with three methods: the immunoassay ELISA and two chromatographic methods (DAD and MS/MS). ELISA is very useful in terms of preliminary screening to detect levels of MCs just around the World Health Organization (WHO) threshold for drinking water (1 µg/L) [51]. However, it does not discriminate between congeners, a very important factor for the proper risk assessment of a CyanoHAB. Indeed, congeners do not have the same toxicity, with intraperitoneal acute LD_50_ spanning between ~50 and 1000 µg/kg bw [2,51]. Therefore, we used ELISA to follow the temporal trend of cyanotoxins and the other two to look at the congeners’ profiles before and after the treatments for a more precise assessment related to the use of allelochemicals for CyanoHAB control (for a detailed discussion about the comparison and suitability of the two chromatographic methods, depending on the aim and the sample, see SM3). Here, we consider data from LC-MS/MS.

The strain from Morocco used here produces a very low amount of MCs in our experimental conditions. The values measured corresponded to a cell quota of about 0.70 fg MCs/cell in the control and MIC, which is about one order of magnitude lower than the lower limit of the range of cell quota values reported for *M. aeruginosa* (1–4000 fg/cell) [2].

*R. aquatilis* extract decreased microcystin concentration. In the MIC, the reduction by 84.5% is consistent with the decrease in cell density, and indeed, the cell quota at the end of the experiment is about the same in the control and in the MIC (0.74 and 0.66 fg/cell). In the MIC/2, the toxin level is reduced by 74.5% vs. a decrease of 25% in cell density, with a cell quota reduced to 1/3 (0.25 fg/cell). Hence, there is no linear concentration-dependent effect, and the MIC/2 seems more effective in cyanotoxin reduction. Having used a monoclonal strain, we can exclude population variability, with nontoxic strains favorably selected by the macrophyte extract, to explain the differences in intracellular MCs in the MIC/2. This could be related to a decrease in *mcy* gene expression (e.g., to save energy used to counteract the oxidative stress) or to an intracellular use of part of the produced MCs, with the same aim. It has been shown that MCs, in case of oxidative stress induced by H_2_O_2_ levels similar to those possibly occurring in a lake surface, can bind the thiol part of proteins involved in photosynthesis to protect them from oxidation [69]. The results in the positive control showed an even lower cell quota than in the MIC/2, but in this case, the determined concentrations of MCs were very low, and the resulting cell quota could be affected by a higher variability and uncertainty.

The analysis of the different MCs congeners showed that the exposure to the *R. aquatilis* extract did not stimulate significant shifts towards more toxic variants so that values determined with the ELISA in the different treatments are comparable in terms of toxicity equivalent.

The little data available show a highly variable situation regarding the effects of allelochemicals on toxin production in relation to cell density. Mecina et al. [24,70] exposed *M. aeruginosa* to barley straw, *Tridax procumbens* and some subfractions of the plants and observed a decrease in total MC-LReq between 2 and 60% vs. a decrease in cells between 0 and 100%, with no correlations between the two parameters. Jang et al. [25] indicated a negative effect of direct exposure to the macrophyte *Lemna japonica* on the density of two strains of *M. aeruginosa*, with a more than threefold increase in the production of MCs. It has also been reported that high doses of pyrogallol, one important polyphenol extracted from macrophytes, caused a significant increase of *mcy* gene transcription and extracellular microcystin concentration in *M. aeruginosa* culture [19]. Variations in toxin production do not depend only on the macrophyte species or allelopathic compound concentrations and are not a direct response to the stress, but they depend on the cyanobacterial strain, on its toxicity and on the complex combination of mechanisms of defense against the specific allelochemicals.

Indeed, our preliminary tests on other species’ density and toxicity show that the response to *R. aquatilis* extract can be very different and cannot be generalized to cyanobacteria other than *Microcystis aeruginosa*. In the other species we tested, we could observe a detrimental effect on cell density and a reduced growth rate, but despite the lower number of cells, the final cyanotoxin concentration was higher or equal to the control level. The cell quota observed in all these strains is in the range of the data observed for these species [2,71,72], except for *C. ovalisporum* treated with the extract. However, most of the studies are related to the intracellular content of the cells and do not take into account the extracellular quantity that in cylindrospermopsin-producing species can be a percentage as high as 80% of toxin [3], therefore underestimating the total toxin produced. Considering also the results of Jang et al. [25] on *M. aeruginosa* previously reported, it is possible that the extract strongly stimulated the production of cylindrospermopsin from the beginning of the experiment, and we measured mostly the extracellular fraction released by the cells dying during the experiment. These results, although preliminary, confirm the need to determine in advance the species-specific response to any allelochemical intended for use in controlling CyanoHABs.

Another result, representing a very relevant aspect in the management of toxic blooms, is that almost the total amount of MCs detected in *M. aeruginosa* are intracellular, except in the positive control, due to cell lysis caused by copper sulfate. The risk of controlling a toxic cyanobacterial bloom is the release of the toxins from the lysed cells by a disinfectant, such as H_2_O_2_ or copper sulfate, as removing dissolved toxins can be very expensive. With such a slightly toxic strain, it is also possible that in the MIC and MIC/2 conditions, the few MC-LR released at the beginning of the experiment due to very few cell lysis are consumed by a very active bacterial community.

The results of ectoenzymatic activities show that the growth reduction of *Microcystis* is not due to the decrease of phosphatase activity, as it actually significantly increases in the MIC and the MIC/2, contrary to what has been suggested [73]. The phosphatase activity is equally attributed to algae and heterotrophic bacteria and is fundamental to recycle the organic phosphorus, especially in conditions of phosphate depletion [23]. Phenolic compounds, the ones that are supposed to be active against algae and cyanobacteria, inhibit phosphatase activity and ectoenzymatic activities in general by binding to proteins and precipitate them [23,73]. However, this process is regulated in a very complex way by multiple factors, such as the ionic composition of the medium, the concentration of the enzymes and the concentrations of phenolic compounds.

With regards to the other activities, they are related to heterotrophic bacteria and are fundamental in maintaining the biogeochemical equilibrium in natural systems. Other studies investigated the effects of eucalyptus and *Spartina alterniflora* extracts on the bacterial density and community composition, showing a negligible effect on such an important component of biota [15,74]. We looked directly at their functional role and demonstrated that in this case, except in the treatment with CuSO_4_, the density and enzymes are actually stimulated by the presence of the macrophyte extract. The inducible glucosidases and chitinases slowly degrade the recalcitrant humic substances that are often dominant in shallow and small lakes, and the dramatic increase observed in these activities after the introduction of the aqueous extract is probably due to the increased availability of these substances.

The analysis of bacteria density and activity helped us in identifying important differences in the effects of copper sulfate and macrophyte aqueous extracts. The former is an aspecific herbicide that destroys algal cells, as well as the bacteria community, therefore undermining the base of a natural system. The latter behaves as a more specific compound whose target is basically the competing algae of the producing macrophyte and, as such, is a promising remediation tool.

## 5. Conclusions

Currently, more than 75% of the drinking water in Morocco is produced from surface water, mainly from dam reservoirs, highlighting the relevance of controlling CyanoHABs. Our study demonstrates that Moroccan macrophyte aqueous extracts are able to inhibit *M. aeruginosa* growth, with the *R. aquatilis* extract revealing the lowest minimum inhibitory concentration. MCs were also reduced and detected only inside the cells, an aspect that can be relevant in the management of toxic blooms, especially when used for drinking water. Considering the nonlinear dependence between aqueous extract concentration and the effects observed on *Microcystis*, *R. aquatilis* could be preferentially used at concentrations lower than the MIC. Finally, the *R. aquatilis* extract had little or no detrimental effect on associated bacteria. All these findings make this aqueous extract a possible good candidate as a remediation measure against *Microcystis* sp.

However, comparison of results obtained with other *Microcystis* strains suggests that variations in toxin production do not depend only on the extract concentrations and are not a direct response to the stress, but they depend on the toxicity of the strain and on the complex combination of mechanisms of defense against the specific allelochemicals. Moreover, our tests on other species’ density and toxicity show that the response to *R. aquatilis* extract cannot be generalized to all cyanobacteria. These results show the need to preliminarily determine the species-specific response to any allelochemical intended for use in controlling CyanoHABs.

Despite these very promising results, it will be necessary to characterize the composition of the extracts to test the activity of various allelochemicals present in the extracts to understand the actual inhibitory agent(s) and to test the toxicity for other aquatic organisms, their biodegradation potential and/or the feasibility of removal from water prior to human consumption, possibly by conducting on-site pilot experiments to avoid secondary pollution before the application in situ.

## Figures and Tables

**Table 1:** Effect of *R. aquatilis* aqueous extracts on intracellular and cell quota MCs at T final.

Treatments	ELISA (µg/L)	LC-MS/MS (µg/L)	HPLC-DAD (µg/L)	Cell Quota (fg/cell)
**NC**	10.20 (±6.68)	15.75 (±4.37)	14.40	0.74 (±0.43)
**PC**	0.22 (±0.01) ***	0.08 (±0.06) ***	5.40	0.11 (±0.09) *
**MIC**	1.58 (±0.74) ***	2.27 (±0.24) **	22.06	0.66 (±0.13)
**MIC/2**	3.79 (±1.25) *	4.01 (±0.44) *	22.04	0.25 (±0.04) *

Each value is the mean ± SD of three replicates. In ELISA, the values are expressed as MC-LReq. In LC-MS/MS and HPLC-DAD, the values are the sum of the different congeners detected. Cell quotas are based on results from LC-MS/MS. * *p* < 0.05, ** *p* < 0.01 and *** *p* < 0.001 indicate significant differences compared to the NC.

**Table 2:** Cell density and total cyanotoxins concentration at T final in experiment with other cyanobacteria.

	Cell Density (cell/L∙10^9^)	Growth Rate (d^−1^)	CTX (µg/L)	Cell Quota fg MC-LReq/cell
**CS1101 C**	1.95 (±0.28)	0.17	82.4 (±10.7) ^1^	39.66 (±1.57)
**CS1101 MIC**	0.11 (±0.02)	−0.19	35.9 (±11.7) ^1^	220.13 (±71.41)
**CS1034 C**	0.29 (±0.03)	0.08	97.1 (±37.8) ^1^	335.66 (±112.34)
**CS1034 MIC**	0.06 (±0.00)	−0.30	80.5 (±33.7) ^1^	1304.50 (±580.85)
**F10 C**	2.20 (±0.33)	0.14	109.2 (±10.1) ^2^	48.41 (±1.75)
**F10 MIC**	4.92 (±0.93)	0.07	275.9 (±41.4) ^2^	57.86 (±22.81)

CS1101 *R. raciborskii*, CS1034 *C. ovalisporum*, F10 *P. rubescens*. C = control, MIC = treated systems. Average of three replicates ± SD. 1 = cylindrospermopsin; 2 = MC-LReq.

## Supplementary Materials

The following are available online at https://www.mdpi.com/article/10.3390/microorganisms9081782/s1, Figure S1: General structure of microcystins, Figure S2: Anti-cyanobacterial activity of the freshwater and marine macrophytes aqueous extracts against *M. aeruginosa* on BG 11 agar plate, Figure S3: Effect of *R. aquatilis* aqueous extracts on MC-congener intracellular concentration at T0 and T final of the experiment, Figure S4: (a) Chl-a cell quota, (b) pheophytin cell quota, (c) carotenoids cell quota, Table S1: Properties of tested MCs congeners, Table S2: Inhibition-zone diameters, minimal inhibitory concentrations (MIC) and minimal algicidal concentrations (MAC) of freshwater and marine macrophytes aqueous extracts.
