# Supplementary material for: Remediation Strategies to Control Toxic Cyanobacterial Blooms: Effects of Macrophyte Aqueous Extracts on Microcystis aeruginosa (Growth, Toxin Production and Oxidative Stress Response) and on Bacterial Ectoenzymatic Activities
Source: Microorganisms. 2021 Aug 23;9(8):1782. doi: 10.3390/microorganisms9081782 (PMC8400474; doi:10.3390/microorganisms9081782)
Supplement: Supplementary file 1 [file microorganisms-09-01782-s001.zip › microorganisms-1311553-supplementary.pdf]

Remediation strategies to control toxic cyanobacteria blooms: effects of macrophyte aqueous extracts on *Microcystis aeruginosa* (growth, toxin production and oxidative stress response) and on bacterial ecto-enzymatic activities

Zakaria Tazart[a,b], Maura Manganelli[a*], Simona Scardala[a], Franca M. Buratti[a], Federica Nigro di Gregorio[a], Mountasser Douma[c], Khadija Mouhri[b], Emanuela Testai[a] and Mohammed Loudiki[b]

Supplementary material

1. Materials and methods

1.1. Comparison of anti-cyanobacterial activity of different macrophyte extracts

1.1.1. Anti-cyanobacterial bioassays

A modified double-layer agar plate method was used to evaluate the anti-cyanobacterial activity of macrophyte aqueous extracts according to Luo et al., (2013). Briefly, soft-agar medium made of 2 mL of cyanobacterial cell suspension at the exponential growth phase (2 x 10^9 cell/L, approximately OD600 = 1.798 at 1 cm) and 3 mL of 1% agar BG11 medium (Fluka, Sigma-Aldrich) was poured onto 20 mL of basal 2% agar BG11. Paper disks of 12 mm, soaked with the extracts at a concentration of 100 mg/mL and copper sulfate (10 mg/mL) used as positive control, were placed onto the center of the plates, immediately put at 4°C for 2 hours. The double-layer agar plates were cultivated for 10 days at 25 °C at a light intensity of 2500 lux and 15:9 (light/dark). The anti-cyanobacterial activity was expressed as mm of a clear zone around the paper disk on the double-layer agar plates.

1.1.2. Determination of the Minimum Inhibitory Concentration (MIC) and Minimum Algicidal Concentration (MAC)

The determination of the MIC of the extracts was carried out in a 96-well microplate using the microdilution assay according to Tazart et al., (2020). Briefly, 150 µL of M. aeruginosa culture in exponential growth phase (2 x 10^9 cells/L) were added to each microplate well. The macrophyte extracts were dissolved in BG11 medium, the same used for *Microcystis* growth, then filtered with a sterile filter (0.22 µm) to remove residues and bacterial contamination, and added to the tested culture to obtain final concentrations of 100 to 0.78 mg/mL for the macrophyte extracts and 0.100 to 0.00078 mg/mL of copper sulfate. Subsequently, the prepared microplates were incubated for 5 days under the described controlled conditions in the thermostat. In order to determine the MAC, 150 µL from each well where the growth was visually inhibited after 5 days of incubation were transferred into new BG11 and incubated for 5 more days under the same conditions described above.

1.2. Allelopathic effect of the most active aqueous extract on M. aeruginosa growth, physiology and toxicity

1.2.1. Measurement of superoxide dismutase (SOD) and catalase (CAT) activities and malondialdehyde (MDA) concentration

The samples for the antioxidant enzymes were prepared according to Li et al., (2016). Every two days, 2 mL of each *M. aeruginosa* culture were centrifuged at 4000xg for 10 minutes at 4ºC. The pellet was resuspended in 0.1M phosphate buffer (pH 6.5) containing 1% (w/v) polyvinylpyrrolidone (PVP) put in an ice bath and then sonicated for 5 minutes in order to disrupt the cells. The sample was then centrifuged at 4000xg for 10 minutes at 4ºC. The supernatant was used for total protein measurement and antioxidant enzyme activity assays. Total protein concentration was determined by the Bradford method (1976), using bovine serum albumin (BSA) as a standard. SOD activity was assayed according to Beauchamp and Fridovich (1971). The absorbance was measured at 560 nm after a 20-minute irradiance of 40–60 mmol photons m^{-2} s^{-1}. One unit of SOD activity was defined as the amount of enzyme that inhibited 50% of photochemical reduction of nitro blue tetrazolium chloride. CAT activity was assayed according to the method of Rao et al., (1996). The reaction mixture contained 1 mL H2O2 (0.3%), 1 mL H2O and 1 mL crude enzyme, and the absorbance was measured at 240 nm. One unit of CAT was defined as the amount of enzyme catalyzing the elimination of 1 mM H2O2 per minute.

MDA content, used as a proxy to measure lipid peroxidation levels, was determined colorimetrically (Esterbauer, H. and K. H. Cheeseman, 1990). 0.5 mL of culture samples were collected every two days and centrifuged at 4000 g for 20 minutes at 4°C. The cell pellets were homogenized with 2 mL of 10% (w/v) trichloroacetic acid (TCA), vortexed and centrifuged again at 4000 g for 10 minutes at 4°C. After centrifugation, 2 mL of the supernatant was mixed with 2 mL of 0.6% thiobarbituric acid (TBA) and heated in a boiling water bath for 15 minutes. The reaction was stopped by transferring the reaction tubes to an ice bath. After cooling, the samples were centrifuged at 4000 g for 10 minutes at 4°C. The absorbance of the supernatant was measured at 532, 600 and 450 nm, using a mixture of 2 mL of MilliQ and 2 mL 0.6% TBA as reference. The MDA level (limit of detection 0.05 µmol/L) was calculated according to the following equation:

$$MDA\ (\mu mol/L) = 6.45 \times OD532 - OD600 - 0.56 \times OD450$$

1.2.2. Cyanotoxins analysis

Microcystins are a group of cyclic heptapeptide (7 amino acids) hepatotoxins produced by a number of cyanobacterial genera, the most notable of which is the widespread Microcystis from which the toxins take their name.

To date, more than 250 different MCs have been identified, with molecular weights in the range of 882–1101Da (WHO, 2020). MCs share a common cyclic heptapeptide structure (Fig. 1) of cyclo-(D-Ala(1)-X(2)-D-Masp(3)-Z(4)-Adda(5)-D-Glu(6)-Mdha(7)) in which X and Z are variable L-amino acids, D-Masp is D-erythro-β-methyl-isoaspartic acid, Mdha is N-methyldehydroalanine and Adda, characteristic moiety of MCs that occurs exclusively in these cyanobacterial peptides, is (2S,3S,4E,6E,8S,9S)-3-amino-9-methoxy-2,6,8-trimethyl-10-phenyldeca-4,6-dienoic acid.

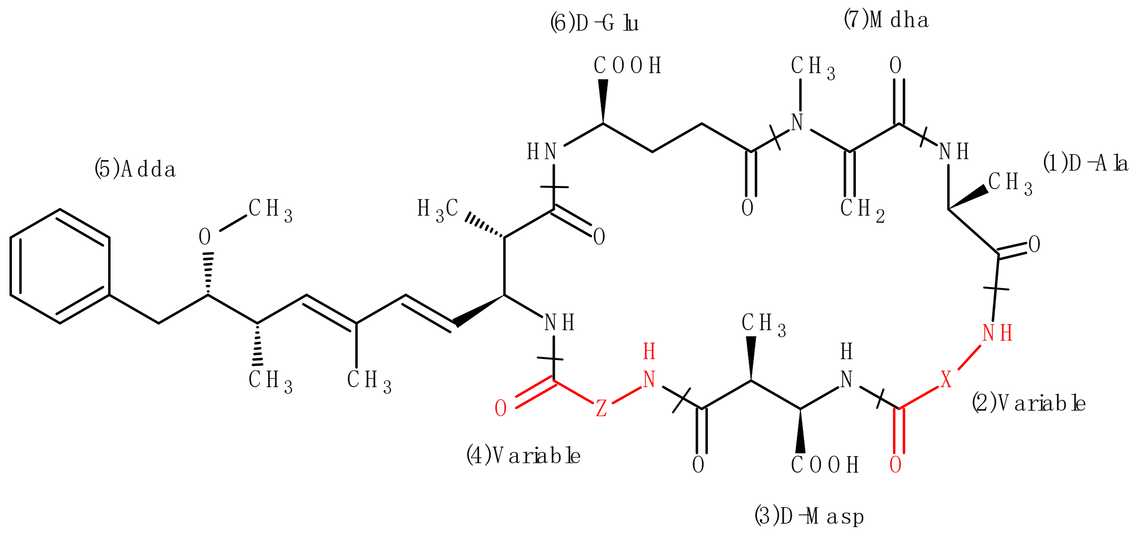

Figure S1. General structure of microcystins

Most frequent structural variations are substitution of L-amino acids at positions 2 and 4, and MCs' nomenclature is based on L-amino acids in these positions for example, using the standard single letter codes for amino acids, MC-LR contains L-leucine (L) at position 2 and L-arginine (R) at position 4.

Other variants are caracterized by minor modifications such as methylation, desmethylation and, in these cases, variations in the molecule are suffixed to the respective variant; for example, [D-Asp3]-MC-LR lacks the methyl group at position 3.

In table 1 the known properties of the tested MCs congeners are reported.

Table S1. Properties of tested MCs congeners

Property	MC-LR	MC-YR	MC-RR	MC-LA	MC-LW	MC-LF	MC-LY	MC-WR	[D-Asp3]-MC-RR	[D-Asp3]-MC-LR	MC-HtyR	MC–HilR
CAS number	101043-37-2	101064-48-6	111755-37-4	96180-79-9	157622-02-1	154037-70-4	123304-10-9	138234-58-9	202120-08-9	120011-66-7	Not assigned	Not assigned
Chemical formula	C49H74N10O12	C52H72N10O12	C49H74N10O13	C46H67N7O12	C54H72N8O12	C51H70N7O12	C52H71N7O13	C54H73N11O12	C48H73N13O12	C48H72N10O12	C53H76N10O14	C50H76N10O12
Molecular weight (g/mol)	995.2	1045.2	1038.2	910.1	1025.2	986.2	1002.2	1068.3	1024.2	981.2	1077.2	1009.2
Z (2)	Leu	Tyr	Arg	Leu	Leu	Leu	Leu	Trp	Arg	Leu	Hty	Hil
X (4)	Arg	Arg	Arg	Ala	Trp	Phe	Tyr	Arg	Arg	Arg	Arg	Arg
Other structural variation	-	-	-	-	-	-	-	-	desmethylation on position (3)	desmethylation on position (3)	-	-

For HPLC-DAD a Perkin-Elmer Series 200 Liquid Chromatograph, equipped with a Perkin-Elmer diode array LC 235 detector and a Restek Pinnacle C-18 DB reversed-phase column (length =25 cm, diameter =4.6 mm) was used. Analyses were carried out using an isocratic method with MeOH:H2O (both containing 0.05 % trifluoroacetic acid (TFA) 70:30 (v/v) (flow 1 ml/min and detection at λ = 238 nm). Before injection all samples were diluted in a 1:1 (v/v) solution of MeOH:Na-phosphate buffer (pH 7) to improve the signal in DAD detection. The MC quantification (for the following variants MCLR, MCLW, MCRR, MCYR, MCLF) was carried out using commercial standard to build a calibration curve prepared with at least five or seven concentrations tested within the range 0.5-100 µM (R2 were >0.99 for all tested congeners) (Buratti et al., 2013).

For LC-MS/MS, separation and identification of the MC variants (MC-RR, MC-YR, MC-LR, MC-LA, MC-LW, MC-LF, MC-LY, [D-Asp3]-MC-RR, [D-Asp3]-MC-LR, MC-HtyR, MC–HilR, MC-WR,) were performed with a liquid chromatographic apparatus consisting of an Ultimate 3000 HPLC system

(Dionex Corporation, Sunnyvale, CA, USA), equipped with an Alltima C18 (2.1 × 250 mm, ID 5 μm) (Alltech, Sedriano, Italy) column thermostated at 40°C. The HPLC module was interfaced by a Turbo Ion Spray source to a triple-quadrupole mass spectrometer API 3000 (Applied Biosystems, Darmstadt, Germany), operating in electrospray ion source (ESI) positive mode.

Quantification of the detected MCs was performed by external standard procedure, referring to calibration straight lines (correlation coefficient R2 = 0.97-0.99). The limit of quantification was 5-25 pg injected in column (Nigro Di Gregorio et al., 2017).

2. Results

2.1. Anticyanobacterial activty of different macrophyte aqueous extracts

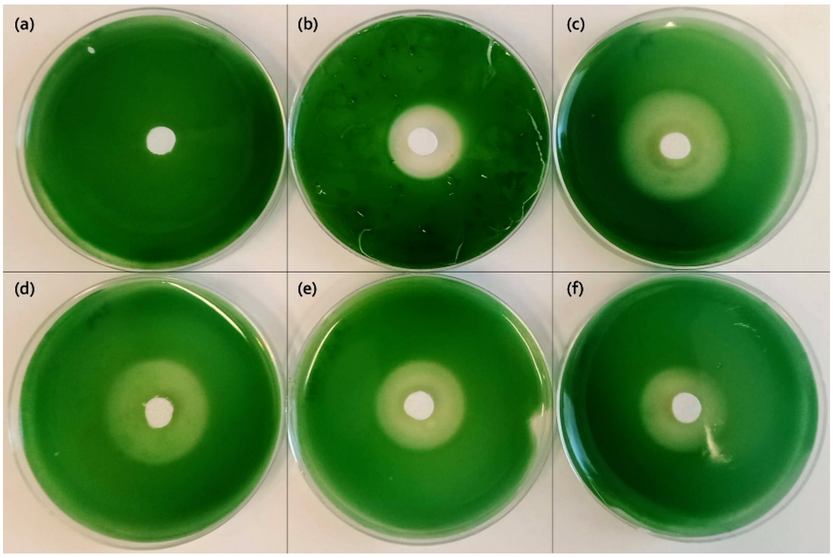

Figure S2. Anti-cyanobacterial activity of the freshwater and marine macrophytes aqueous extracts against *M. aeruginosa* on BG 11 agar plate: (a) negative control, (b) copper sulfate, (c) *R. aquatilis*, (d) *P. natans*, (e) *S. lacustris* and (f) *C. nodosa*

The agar disk diffusion is a qualitative method, as it is impossible to quantify the amount of the antimicrobial agent diffused into the agar medium. The clear area surrounding the disk in the plates with the extracts is not completely transparent as in the positive control, probably due to the diffusion of a concentration of the extracts lower than the minimum inhibitory concentration, that allowed few cells to grow. However antibacterial activity was evident and significant.

Table S2. Inhibition-zone diameters, minimal inhibitory concentrations (MIC) and minimal algicidal concentrations (MAC) of freshwater and marine macrophyte aqueous extracts

Treatments	Inhibition diameter[1] (mm)	MIC[2] mg/mL	MAC[2] mg/mL
Negative control	NA	NA	NA
Copper Sulfate	22 ± 0.88 ***	0.003	0.006
Ranunculus aquatilis	36 ± 2 ***	13.00	25.00
Potamogeton natans	30 ± 2 ***	25.00	50.00
Scirpus lacustris	31 ± 1 ***	50.00	100.00
Cymodocea nodosa	32 ± 1 ***	25.00	100.00

Each value is the mean ± SD of three replicates, *** $p < 0.001$ indicate significant differences between treatments groups and negative control, NA=non-active. 1=disks were soaked with aqueous extracts at a concentration of 100 mg/mL and with copper sulfate at 10 mg/mL.

2=concentrations tested ranged between from 0.078·10^{-2} to 0.100 mg/mL for copper sulfate and 100.00-0.78 mg/mL for the aqueous extracts

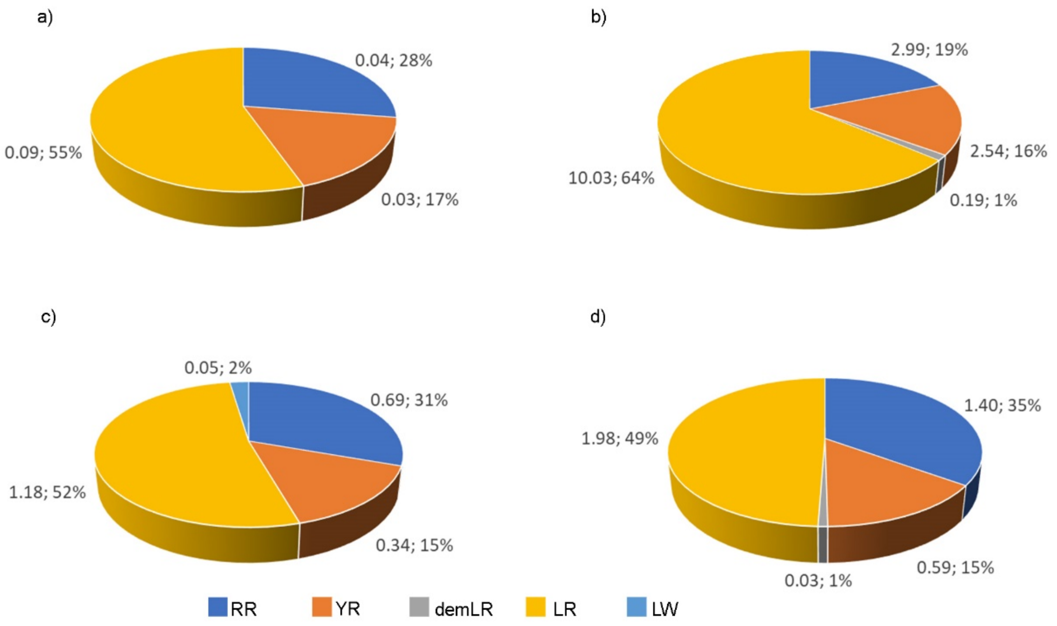

Figure S3. Effect of *R. aquatilis* aqueous extracts on MC-congener intracellular concentration at T0 and T final of the experiment. (a) control at T0, (b) control at T final, (c) MIC at T final and (d) MIC/2 at T final. Each value is the mean of three replicates

3. Discussion

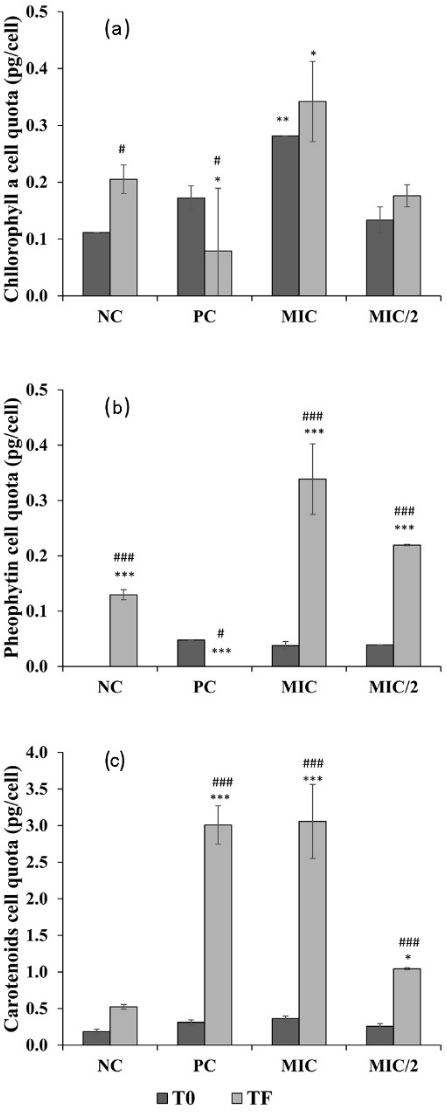

Figure S4. (a) Chl-a cell quota, (b) pheophytin cell quota, (c) carotenoids cell quota. *p<0.05, **p<0.01 and***p< 0.001 indicate significant differences compared to the negative control in the T0 or TF and #p<0.05, ##p<0.01 and ###p< 0.001 indicate significant differences between time for each treatment group

3.1. Analytical methods

LC-MS/MS is more sensitive and more specific in the identification of the different congeners, and it is the suggested method for the purposes of research and risk assessment, but it is not available in many of the laboratories that have to control the toxicity of a bloom. The availability of HPLC-DAD equipment and expertise is more common, especially in some low-income countries, although its sensitivity and specificity is lower than LC-MS/MS. We noticed that results were in good agreement among the two analytical methods only in the control, where the culture media did not interfere with MC detection and the concentrations were high enough for a reliable quantification. In order to detect MCs, treated samples had to be concentrated more than 100x (vs 10x in the LC-MS/MS), hence concentrating macrophyte aqueous extract components as well. This made the background noise quite high, very likely interfering with the resolution and consequently possibly causing an overestimation of the peaks (further amplified by the concentration factor). Therefore, while HPLC-DAD can still be a very valuable tool to detect MC variants in clean water samples,

whenever more 'dirty' samples (as the one used here) have to be tested, a selective extraction method should be used to purify the samples, followed by a measurement of the recovery, in order to have reliable data.

References

Beauchamp, C., Fridovich, I., 1971. Superoxide dismutase: Improved assays and an assay applicable to acrylamide gels. Anal. Biochem. 44, 276–287. https://doi.org/10.1016/0003-2697(71)90370-8.

Bradford, M.M., 1976. A rapid and sensitive method for the quantitation of microgram quantities of protein utilizing the principle of protein-dye binding. Anal. Biochem. 72, 248–254. https://doi.org/10.1016/0003-2697(76)90527-3.

Buratti, F.M., Scardala, S., Funari, E., Testai, E., 2013. The conjugation of microcystin-RR by human recombinant GSTs and hepatic cytosol. Toxicol. Lett. 219, 231–238.

Esterbauer, H. and K. H. Cheeseman (1990). [42] Determination of aldehydic lipid peroxidation products: Malonalde-hyde and 4-hydroxynonenal. Methods in Enzymology, Academic Press. 186: 407-421.

Li, J., Liu, Y., Zhang, P., Zeng, G., Cai, X., Liu, S., Yin, Y., Hu, X., Hu, X., Tan, X., 2016. Growth inhibition and oxidative damage of *Microcystis aeruginosa* induced by crude extract of *Sagittaria trifolia* tubers. J. Environ. Sci. (China) 43, 40–47. https://doi.org/10.1016/j.jes.2015.08.020.

Luo, J., Wang, Y., Tang, S., Liang, J., Lin, W., Luo, L., 2013. Isolation and Identification of Algicidal Compound from Streptomyces and Algicidal Mechanism to *Microcystis aeruginosa*. PLoS One 8, 1–14. https://doi.org/10.1371/journal.pone.0076444.

Nigro Di Gregorio, F., Bogialli, S., Ferretti, E., Lucentini, L., 2017. First evidence of MC-HtyR associated to a *Plankthothrix rubescens* blooming in an Italian lake based on a LC-MS method for routinely analysis of twelve microcystins in fresh-waters. Microchem. J. 130, 329–335.

Rao, M. V., Paliyath, G., Ormrod, D.P., 1996. Ultraviolet-B- and Ozone-Induced Biochemical Changes in Antioxidant En-zymes of *Arabidopsis thaliana*. Plant Physiol. 110, 125–136. https://doi.org/10.1104/pp.110.1.125.

Tazart, Z., Douma, M., Caldeira, A.T., Tebaa, L., Mouhri, K., Loudiki, M., 2020. Highlighting of the antialgal activity of organic extracts of Moroccan macrophytes: potential use in cyanobacteria blooms control. Environ. Sci. Pollut. Res. 27, 19630–19637. https://doi.org/10.1007/s11356-020-08440-w.

World Health Organization. (2020) .Cyanobacterial toxins :microcystins .World Health Organization .https//:apps.who.int/iris/handle/10665/338066. License: CC BY-NC-SA 3.0 IGO. WHO/HEP/ECH/WSH/2020.6.
